# Transient systolic anterior motion with junctional rhythm after mitral valve repair in the intensive care unit

**DOI:** 10.1186/s13089-018-0111-6

**Published:** 2018-11-12

**Authors:** Yusuke Seino, Nobuo Sato, Kimiya Fukui, Junya Ishikawa, Masahi Nakagawa, Takeshi Nomura

**Affiliations:** Department of Intensive Care Medicine, Tokyo Women’s Medical University, 8-1, Kawada-cho, Shinjuku-ku, Tokyo, Japan

**Keywords:** Systolic anterior motion, Left ventricular outflow tract obstruction, Mitral valve repair, Transesophageal echocardiography

## Abstract

Systolic anterior motion (SAM) after mitral valve repair (MVR) can adversely affect hemodynamics due to exacerbation of left ventricular outflow tract obstruction and mitral regurgitation. Intraoperative transient SAM after MVR can usually be managed with hemodynamic maneuvers under continuous monitoring by transesophageal echocardiography (TEE). However, during postoperative intensive care management, transient SAM is seldom diagnosed and the start of treatment may be delayed. We present a case of transient SAM after MVR with abrupt deterioration due to junctional rhythm in the intensive care unit (ICU). TEE revealed that conversion from normal sinus rhythm into junctional rhythm induced the exacerbation of SAM. TEE was useful for identifying the etiology of unstable hemodynamics after cardiac surgery in the ICU, similar to its use in the operating room.

**Electronic supplementary material:**

The online version of this article (10.1186/s13089-018-0111-6) contains supplementary material, which is available to authorized users.

## Background

Systolic anterior motion (SAM) after mitral valve repair (MVR) can adversely affect hemodynamics due to exacerbation of left ventricular outflow tract obstruction (LVOTO) and mitral regurgitation (MR) [1]. Although some mechanisms are understood, the effect of junctional rhythm on SAM, LVOTO, and MR remains unclear [1, 2]. Intraoperative transient SAM is usually diagnosed with transesophageal echocardiography (TEE), and should immediately be treated with hemodynamic agents under TEE monitoring. However, transient SAM in the ICU due to junctional rhythm without signs of ischemia can be difficult to diagnose. Even in this case, SAM cannot be thought of as the cause of hemodynamic disorder. Only TEE observation after an event can assist in the choice of treatment.

## Case presentation

A 55-year-old man with severe MR and middle scallop prolapse due to torn chordae underwent MVR. Preoperative LV ejection fraction, tricuspid annular plane systolic excursion and right ventricular fractional area change were 62%, 26 mm and 44%, respectively. MVR was performed with folding middle scallop and annuloplasty with a 30-mm Physio II annuloplasty ring (Edwards Lifesciences LLC, Irvine, CA, USA). MR was well controlled. Despite difficulty weaning from cardiopulmonary bypass and some persistent surgical bleeding, he was hemodynamically stabilized on admission to the ICU without evidence of SAM and LVOTO.

He became hemodynamically unstable on the first postoperative day, with parameters as follows: heart rate 87 beats/min (bpm), sinus rhythm, arterial pressure 80/48 mmHg, pulmonary artery pressure 28/20 mmHg, central venous pressure 17 mmHg, cardiac index 1.7 L/min/m^2^, right ventricular stroke work index (RVSWI) 1.6 g/m^2^/beat, and mixed venous oxygen saturation (SvO_2_) 54%.

Transthoracic echocardiography (TTE) was performed to investigate the cause of hemodynamic deterioration; however, adequate images could not be obtained. TEE was then performed and showed right ventricular (RV) dysfunction without SAM and LVOTO. We decided to increase the dose of inotrope (adrenaline) to improve RV function and the hemodynamics stabilized.

Severe hypotension developed on the second postoperative day. Hemodynamic parameters were as follows: heart rate 90 bpm, arterial pressure 50/42 mmHg, pulmonary artery pressure 28/20 mmHg, central venous pressure 13 mmHg, cardiac index 1.6 L/min/m^2^, RVSWI 2.4 g/m^2^/beat, and SvO_2_ 48%. The ventilator settings were as follows: pressure assist-control ventilation, respiratory rate 14 breaths/min, peak inspiratory pressure 16 cmH_2_O, inspiration time 1.6 s, positive end-expiratory pressure 10 cmH_2_O, and fraction of inspiratory oxygen 0.6.

Although the dose of dopamine, dobutamine and adrenaline was greatly increased to 5.4 µg/kg/min, 5.4 µg/kg/min and 0.05 µg/kg/min, respectively, and intra-aortic balloon pumping was started, hemodynamic parameters did not show improvement. No pulmonary vasodilator was used because of avoiding hypotension.

**Fig. 1:**
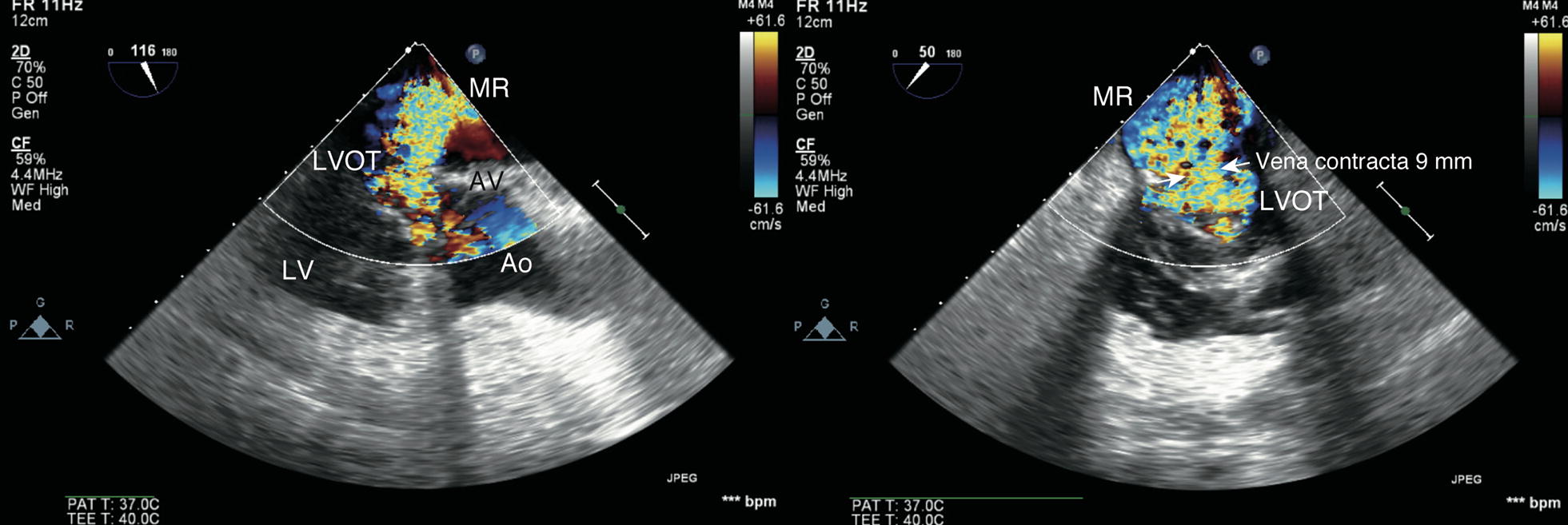
Massive mitral regurgitation and turbulent flow in the left ventricular outflow tract. *MR* mitral regurgitation, *LVOT* left ventricular outflow tract, *Ao* ascending aorta, *AV* aortic valve, *LV* left ventricle

**Fig. 2:**
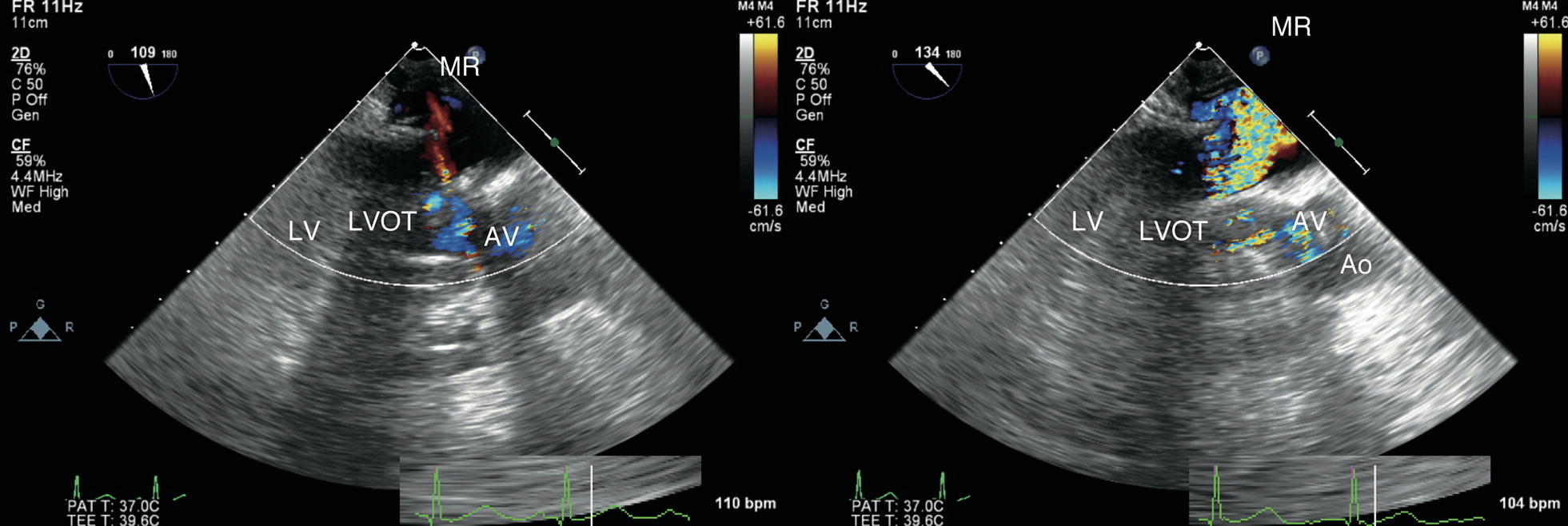
Mitral regurgitation in normal sinus rhythm (left) and junctional rhythm (right). *MR* mitral regurgitation, *LVOT* left ventricular outflow tract, *Ao* ascending aorta, *AV* aortic valve, *LV* left ventricle

**Fig. 3:**
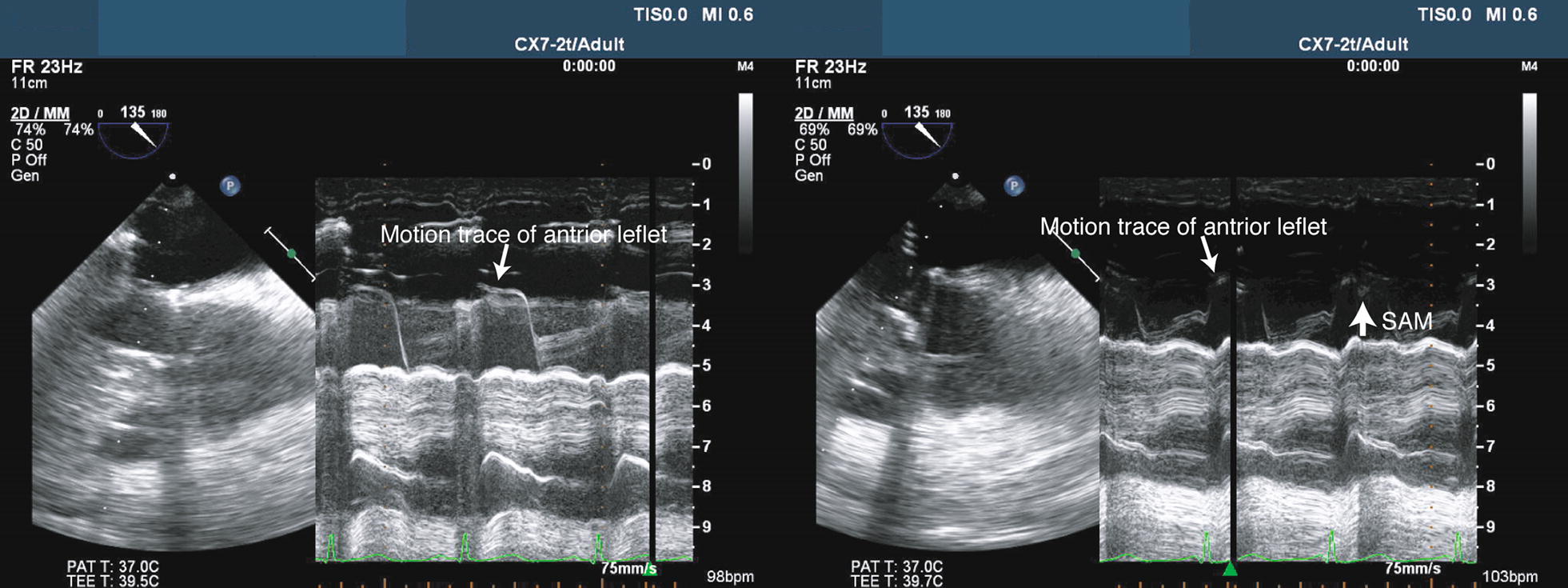
Motion tracing of the anterior leaflet in normal sinus rhythm (left) and junctional rhythm (right). Systolic anterior motion (SAM) is shown in junctional rhythm (large arrow)

**Fig. 4:**
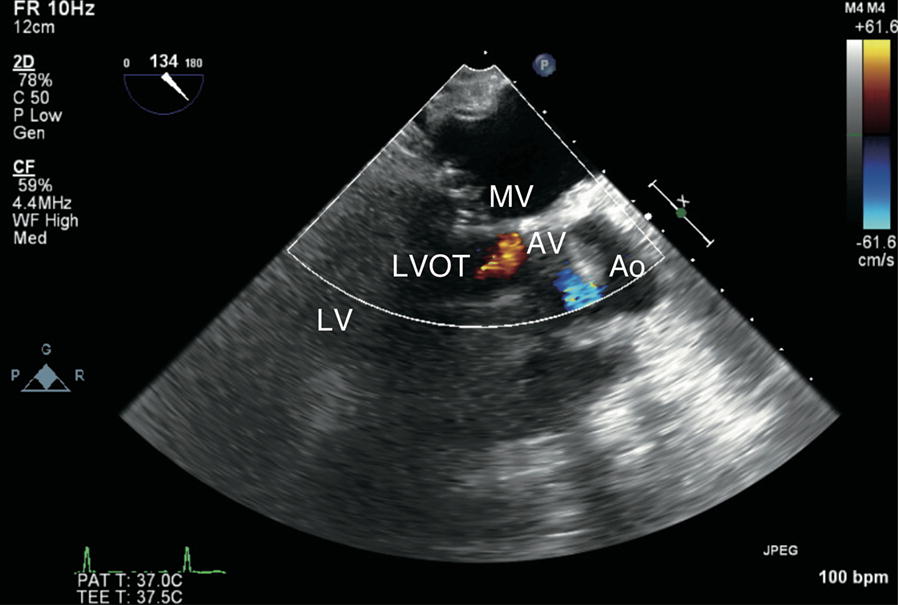
Mid-esophageal long axis view under atrial pacing on the sixth postoperative day. There is no mitral regurgitation and left ventricular outflow tract obstruction. *MV* mitral valve, *LVOT* left ventricular outflow tract, *Ao* ascending aorta, *AV* aortic valve, *LV* left ventricle

Repeat TEE revealed the hyperdynamic left ventricle (LV) without any regional wall motion abnormality, as well as LVOTO and severe MR (vena contracta 8 mm) due to SAM (Fig. 1; Additional file 1). In addition, RV dysfunction was still presented. We immediately reduced the inotrope dose and started vasopressors (vasopressin 2 unit/h) with volume loading (acetate Ringer 500 mL and 5% albumin 250 mL). SAM, LVOTO, and MR rapidly resolved and hemodynamic parameters stabilized. However, when normal sinus rhythm converted into junctional rhythm, SAM, LVOTO and severe MR recurred and hemodynamic parameters again became unstable (Figs. 2, 3; Additional files 2, 3, 4). TEE showed that conversion from normal sinus rhythm into junctional rhythm worsened the severity of SAM, LVOTO, and MR. The electrocardiogram showed junctional rhythm alternating with sinus rhythm, repeated at short intervals. We found that the rhythm change had a significant effect on the emergence of SAM and hemodynamic deterioration. Temporary atrial pacing was used to stabilize the heart rhythm. After starting atrial pacing, the hemodynamics stabilized and SAM, LVOTO, and MR did not recur (Fig. 4). He discharged from ICU on the 22nd postoperative day and from the hospital on the 43rd postoperative day.

## Conclusions

We identified two important clinical issues. Junctional rhythm can cause deterioration of SAM, LVOTO, and MR, and atrial pacing can improve hemodynamics in unstable normal sinus rhythm. TEE is useful for the identification of causes of unstable hemodynamics after cardiac surgery in the ICU as well as in the operating room.

First, junctional rhythm can cause deterioration of SAM, LVOTO, and MR and atrial pacing can improve hemodynamics. Some mechanisms of SAM are related to geometric factors, kinetic factors, and structural abnormalities [3]. Geometric factors include annular undersizing, anterior mitral valve displacement, low anterior:posterior leaflet length ratio, reduced mitro-aortic angle, and distance reduction between the MV coaptation point and interventricular septum [4]. Hyperdynamic LV is a kinetic factor. Structural abnormalities include small LV, chordal anomalies, bulging interventricular septum, papillary muscle displacement, redundant posterior leaflet, and redundant anterior leaflet. Some authors reported that ventricular pacing or atrioventricular sequential pacing can affect the severity of SAM [5, 6]. In the present case, junctional rhythm caused deterioration of SAM. Junctional rhythm causes atrioventricular dyssynchrony and loss of atrial kick. These can contribute to decreases in mean arterial pressure and cardiac output by approximately 15% and the decreases can be accentuated in patients with heart disease [7, 8]. The mechanism by which junctional rhythm causes deterioration of SAM might be the impairment of LV filling due to atrioventricular dyssynchrony and loss of the atrial kick in junctional rhythm. In addition, underlying right ventricular dysfunction also plays a role in LV underfilling. There are some causes of RV dysfunction after cardiac surgery including inadequate myocardial protection during cardiac arrest, myocardial ischemia due to residual air, high pulmonary vascular resistance, and inadequate settings of mechanical ventilation [9]. In the present case, the right cause of RV dysfunction is unclear; however, long CPB, residual air, compression for control of bleeding by the surgeons might be related to RV dysfunction.

The management of SAM depends on the underlying mechanism and severity. In most cases, volume loading, discontinuation or reduction of inotropic drugs, and administration of beta blockers and vasopressors can resolve SAM [10–12]. It has been demonstrated that conservative hemodynamic maneuvers for intraoperative transient SAM are reliable and result in good clinical outcomes, although some cases require surgical correction with second cardiopulmonary bypass [13]. Since the present case had right ventricular failure, maintaining normal sinus rhythm seemed to be more important than in patients without right ventricular failure [14].

Second, TEE in the ICU is useful for the identification of causes of unstable hemodynamics after cardiac surgery. The usefulness of echocardiography for diagnosis and decision-making has been shown in the ICU, and basic echocardiography skills have become mandatory for all intensivists in some guidelines [15–17]. On the other hand, the use of TEE is reserved for instances in which diagnostic information for clinical decision-making cannot be obtained by TTE in a timely manner. However, TEE is considered an advanced skill and is not considered a minimum requirement for intensivists [17–19].

After cardiac surgery, obtaining useful images is sometimes difficult due to the narrowing of acoustic windows caused by the surgical incision, drapes, and drains. In addition, unclear images are insufficient for clinical decision-making in critical situations. Strengths of TEE include less dependence on operator skills and better spatial resolution compared to that of TTE [20]. In the present patient, the causes of recurrent hemodynamic exacerbation were different and could not be diagnosed without TEE. Basic hemodynamic assessment with TEE is not that difficult and training in basic TEE skills with simulators is evolving [21, 22]. Thus, the barrier to learning of basic TEE skills by intensivists can be overcome.

This case report shows that junctional rhythm can cause deterioration of SAM, LVOTO, and MR, and can lead to unstable hemodynamics in a patient with right ventricular failure after MVR. Atrial pacing can resolve SAM, LVOTO, and MR and can improve hemodynamics in a patient with unstable normal sinus rhythm. TEE in the ICU can play a pivotal role in clinical decision-making. Given that there are various causes of hemodynamic deterioration after cardiac surgery and some cannot be diagnosed without TEE, both TTE and basic TEE skills are required for intensivists.

## Additional files

**Additional file 1.** Mid-esophageal long axis view on the second postoperative day. Massive mitral regurgitation and turbulent flow is seen in the left ventricular outflow tract.

**Additional file 2.** Mid-esophageal long axis view in normal sinus rhythm.

**Additional file 3.** Mid-esophageal long axis view in junctional rhythm.

**Additional file 4.** Mid-esophageal long axis view in junctional rhythm (2-dimensional mode).

## Abbreviations

ICU: intensive care unit
LVOTO: left ventricular outflow tract obstruction
MR: mitral regurgitation
MVR: mitral valve repair
RVSWI: right ventricular stroke work index
SAM: systolic anterior motion
SvO_2_: mixed venous oxygen saturation
TEE: transesophageal echocardiography
TTE: transthoracic echocardiography
